# Effect of Core Stability Training Monitored by Rehabilitative Ultrasound Image and Surface Electromyogram in Local Core Muscles of Healthy People

**DOI:** 10.1155/2019/9130959

**Published:** 2019-06-23

**Authors:** Yaochao Zheng, Songjian Ke, Caina Lin, Xiao Li, Cuicui Liu, Yuanyuan Wu, Wenjun Xin, Chao Ma, Shaoling Wu

**Affiliations:** ^1^Rehabilitation Medicine Department, Sun Yat-sen Memorial Hospital, Sun Yat-sen University, Guangzhou, China; ^2^Department of Physiology and Pain Research Center, Guangdong Province Key Laboratory of Brain Function and Disease, Zhongshan Medical School, Sun Yat-sen University, Guangzhou, China

## Abstract

**Background:**

The purpose of this study is to investigate the influence of transverses abdominis and lumbar multifidus thickness activation and electromyogram signal characteristics after core stability training monitored by rehabilitative ultrasound imaging and surface electromyogram.

**Methods:**

60 healthy volunteers were allocated randomly into two groups, one of which received monitoring training and the other participated identical training without monitoring. Ultrasound image and surface electromyogram signal were collected at 0, 4, and 8 weeks during training. The muscle thickness activation ratio value and integrated electromyogram value were then extracted. During the training, the monitoring group was monitored by real-time rehabilitative ultrasound imaging and surface electromyogram while the control group was not.

**Results:**

There are no differences in performance of local core muscles between both groups before training (*p* > 0.05). Compared with the control group, the thickness contraction ratio value and integrated electromyogram value of core muscles in the monitoring group were higher after 8 weeks' training (*p* < 0.05).

**Conclusion:**

Together, the core stability training monitored by rehabilitative ultrasound imaging and surface electromyogram can markedly activate and enhance local core muscles in healthy people, providing a potential strategy to treat low back pain more effectively.

## 1. Introduction

Low back pain (LBP) is a common disease bothering most populations in both developed and developing countries [1, 2]. Various approaches have been applied to treat LBP, including electrical stimulation [3], message [4], acupuncture [5] and invasive surgery [6]. However, no single modality appears to be superior and the effectiveness of these treatments is still questionable and uncertain. In recent years, core stability was defined as the ability to maintain a stable neutral spine position and core stability training has been proved to be useful in the treatment of LBP through decreasing pain [6–8], reducing disability [9], and preventing relapse [10].

Core muscles are categorized into global muscles (namely, the rectus abdominis, erector spinae, and obliquus externus abdominis) and local muscles (namely, transverse abdominis and lumbar multifidus) by function [11]. The global muscles participate in trunk movements, whereas the local muscles play an important role in core stabilization [12]. As local stabilizers, the transverse abdominis (TrA) and lumbar multifidus (MF) play important roles in the functional activities of spine. Our previous study shows that atrophy of the MF and the TrA could be frequently demonstrated in patients with LBP [13]. However, the application of core stability training targeting TrA and MF needs further optimization and innovation because of uncertainty about the best way to apply exercise. The key of effective training is to ensure target core muscles contract in correct patterns.

Many studies have been previously reported on the relationship between the surface electromyogram (surface EMG) and muscle force [14]. The reliability and validity of this measurement are well established. Since it was found that recording the muscle activity of the deep fibers of TrA or MF by surface EMG may be inaccurate, another method currently used to examine core muscles function is rehabilitative ultrasound imaging (RUSI), an up-to-date method used to evaluate muscle activation without invasion [15, 16]. RUSI is increasingly adapted in pain therapy to quantify core muscle performance, assess clinical outcomes, and provide biofeedback during functional re-education [17]. The degree of muscle thickness change that occurs during exercise can be measured by RUSI, whose reliability is well established for younger adults [18].

Above all, the objective of this study is to determine the effect of core stability training monitored by RUSI and surface EMG in local core muscles of healthy people.

## 2. Methods

### 2.1. Experimental Approach to the Problem

To investigate the effect of core stability training monitored by RUSI and surface EMG, two methods were used to monitor core stability training in the monitoring group. The monitoring group received specific core stability training monitored by RUSI and surface EMG, while the control group finished the same training plan without monitoring. Muscle thickness activation ratio and EMG characteristic were measured by RUSI and surface EMG and collected at 0, 4, and 8 weeks during long-term training.

### 2.2. Participants

The participants were recruited in Guangzhou, China, between August and December during 2017. The inclusion criteria were as follows: (1) male; (2) the age of 20–25 years old; (3) the willingness to complete a chosen training plan; and (4) the written informed consent of the participants. Participants were excluded if they have (1) the presence of the herniated lumbar disk or lumbar disk protrusion; (2) the presence of the vertebral fracture(s) or other conditions that needs surgery; (3) cardiovascular or systemic diseases or any condition which contraindicated or made the training impossible; (4) the presence of the psychiatric disorder which might affect the compliance and the evaluation of symptoms; (5) inflammatory, infectious or malignant diseases of the vertebra; and (6) the presence of severe neurological and structural deformity.

To ensure that all criteria were fulfilled, an experienced medical doctor would examine each participant. All participants were informed about the purpose and information of the study, including the randomization process. Before participating in the study, they also received a written informed consent, which was approved by the ethics committee of the authors' hospital. This study was qualified and registered in the Chinese Clinical Trial Registry as ChiCTR1800014609, where our data were collected and recorded. Besides, the study design meets the criteria of the latest version of the Declaration of Helsinki.

### 2.3. Procedures

#### 2.3.1. Randomization

Using a computer-generated random numbers' table, an independent statistician performed all the random allocation of participants. The statistician was not aware of the eligibility of the participants and performed the randomization procedure following the baseline examination of all participants and then informed the participants via messages about group allocation. The randomization codes were stored in a sealed opaque envelope until the study ended.

#### 2.3.2. Intervention

All participants were allocated randomly into the monitoring group and control group, while the control group received the same training without monitoring. All the intervention took place at our sport center.

**Figure 1:**
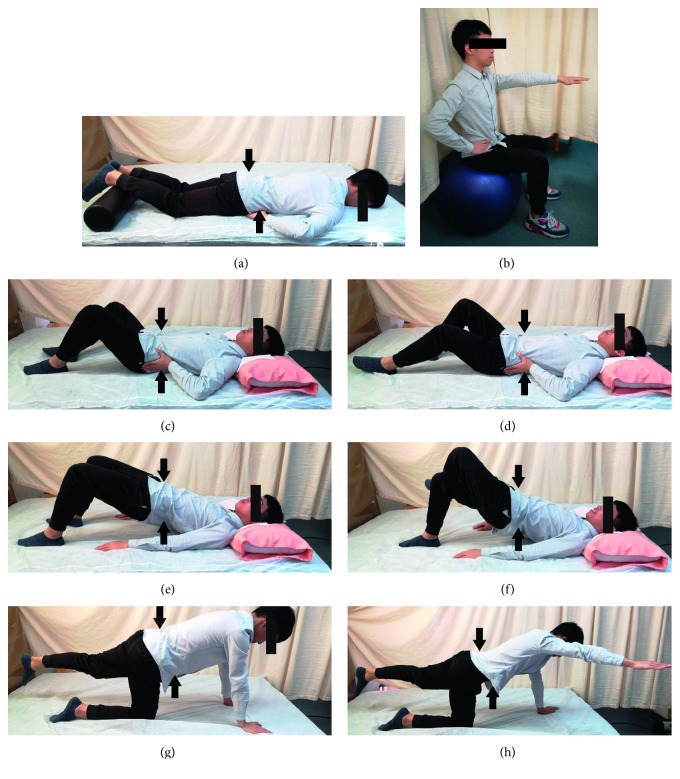
Core stabilization exercise. (a) Train TrA muscle activation in a prone lying position without spinal and pelvic movements for 10 seconds. Keep respiration normal. You gently draw in the lower anterior abdominal wall below the navel level (abdominal drawing-in maneuver) with supplemented contraction of pelvic floor muscles, control your breathing normally, and have no movement of the spine and pelvis while lying prone on a couch with a small pillow placed beneath your ankles. (b) Train MF activation in an upright sitting position. You raise the contralateral arm while performing the abdominal drawing-in maneuver in a sitting position on a yoga ball. (c) Perform cocontraction of the two muscles in a crooked lying position with both hips at 45 degrees and both knees at 90 degrees. Then, you abduct one leg to 45 degrees of hip abduction and hold it for 10 seconds. (d) Train cocontraction of these muscles in a crooked lying position with both hips at 45 degrees and both knees at 90 degrees. Then, you slide a single leg down until the knee is straight, maintain it for 10-second holds, and then slide it back up to the starting position. (e) Perform cocontraction of the two muscles while raising the buttocks off a couch from a crooked lying position until your shoulders, hips, and knees are straight. You sustain this pose for 10 seconds and then lower the buttocks back down to the couch. (f) Train muscle cocontraction while raising the buttocks off a couch from a crooked lying position with one leg crossed over the supporting leg. You raise the buttocks off the couch until the shoulders, hips, and knees are straight. You sustain this pose for 10 seconds and then lower the buttocks back down to the couch. (g) Perform cocontraction while raising a single leg from a four-point kneeling position and keeping your back in a neutral position. You sustain this pose for 10 seconds and then return the leg to the starting position. (h) Train muscle cocontraction while raising an arm and alternate leg from a four-point kneeling position and keeping your back in a neutral position. You sustain this pose for 10 seconds and then return to the starting position. Black arrows show the contraction direction of core muscles.

The physical therapist demonstrated and explained a modified exercise plan based on a previous study [19] to all participants (Figure 1). The participants carried out the core stability exercise program for 45 min, three times a week for 2 months. At the first 4 weeks, the participants were asked to finish primary training, which is shown in Figures 1(a)–1(c), 1(e), and 1(g), and then, in the next four weeks, they should finish superior training shown in Figures 1(a), 1(b), 1(d), 1(f), and 1(h). All the participants were encouraged to finish training 10 repetitions 2 times a day. The program was divided into three categories: warm up, main part, and cool down. In the beginning of each exercise, the examiner determined the participant's lumbar neutral position and the participants were asked to hold this position during training. During training, several certified strength and conditioning coaches guided the participants of both groups to ensure that the training plan was properly executed considering its technique. The time spent on education was need based and varied within both groups and participants. Questions and discussions were encouraged.

During training, rehabilitative ultrasound image and surface EMG were used to provide real-time biofeedback to control the neuromuscular mechanism in the monitoring group while control group were not. In order to give real-time training feedback to participants and guarantee the correct training, four groups of muscles were monitored, including rectus abdominis, erector spinae, TrA, and MF. Monitoring was maintained throughout the whole training once the participants started exercise. When training, the activities of the rectus abdominis and erector spinae were not allowed to be more intense than those of the TrA and MF.

Through shaving, abrading, and cleansing with alcohol, the skin was carefully prepared to reduce skin impedance below 4 kΩ. Once the skin was dry, bipolar self-adhesive, pregelled Ag/AgCl surface electrodes were positioned at an interelectrode distance of 2 cm to the following locations. The electrode placement for rectus abdominis was placed 2 cm lateral to the umbilicus. The TrA electrode was positioned 1.5 cm anterior to the midaxillary line, near the ultrasound site. The erector spinae electrode was set at 5 cm lateral to the L3 spinous process. The MF electrode was positioned 1 cm lateral to the L4 spinous process. All the reference electrode placements were over the anterior superior iliac spine (ASIS). And, the RUSI monitoring sites of each muscle are as follows. The monitoring site of rectus abdominis was set at 3 cm lateral to the umbilicus. The monitoring site of TrA was positioned 2.5 cm anterior to the midaxillary line, at the midpoint between the inferior rib and the iliac crest. The monitoring site of erector spinae electrode was positioned 7 cm lateral to the L3 spinous process, while the monitoring site of MF electrode was positioned 2 cm lateral to the L4 spinous process.

#### 2.3.3. Outcome Measures

All muscle thicknesses and EMG characteristics were carried out at 0, 4, and 8 weeks during study. The measurement positions of TrA and MF were the same as the monitoring sites.

Using B-mode ultrasound imaging (CHISON Q9, China) with transducers with a range of 5–8 MHz, muscle thickness was measured. Several studies have proved the high reliability of RUSI in measuring the thickness of trunk muscles [20, 21]. As core muscle thickness changes during expiration [22], recordings were made at the end of relaxed expiration during the measurements of TrA and MF thickness. The transducer was held perpendicular to the skin surface with a minimum pressure required to achieve a clear image. To improve acoustic coupling, a water-soluble transmission gel was placed over the scan head. The average thickness of three trials was calculated. Activation ratio = relax thickness/contraction thickness.

The EMG data was collected using the TeleMyo2400T (Noraxon, USA). The raw EMG signals were processed using MyoResearch software (Noraxon, USA) at a sampling frequency of 1500 Hz with band-pass filtering at 15–500 Hz for a noise reduction associated with electrical interference. EMG data were processed in MATLAB (MathWorks, Inc., Natick, MA, USA). Each muscle's EMG data were high-pass filtered (40 Hz, 4th order Butterworth), rectified, and low-pass filtered (40 Hz, 4th order Butterworth) to calculate the linear envelope describing muscle activation. All EMG data were measured for 3 s, discarding the first and last “s.” The average IEMG value of the three trials was calculated.

### 2.4. Statistical Analysis

All data are recorded and displayed as mean ± standard deviation (SD). Statistical analysis was conducted using the SPSS 20.0 software (IBM, USA). All the outcome variables were analyzed on the intention-to-treat principle and examined by the normality test firstly. The demographic data were examined by descriptive statistics. Sphericity assumption was identified by the test, and the differences of all the variables in each group were compared using the ANOVA for repeated measures. If the interactive effect of time and group exists, the independent samples *t*-test between groups were carried out. We also performed the paired samples *t*-test to determine the differences in activation ratio and integrated EMG (IEMG) at 0, 4, and 8 weeks. The significance level was set at *p* < 0.05 for all of these tests.

## 3. Results

### 3.1. Sample Characteristics

60 participants were recruited in this study, and each group consists of 30 participants. All participants are male. There is no significant difference between groups in age, height, weight, BMI, culture level, and daily amount of exercise (Table 1). This implies that the groups were similar before treatment and the changes observed following the procedure can be referred to the monitor effect on the muscles. All the data are available.

### 3.2. RUSI Data on Muscle Thickness Activation Ratio

All variables meet the normal distribution. Sphericity assumptions in both sides were identified by the test (*p*=0.424 (left) and *p*=0.165 (right) for TrA; *p*=0.660 (left) and *p*=0.127 (right) for MF), and the ANOVA for repeated measures can be used to perform the data analysis. The results show that the interaction between treatment effects and time effects was significant (for TrA, *F* = 18.378, *p* ≤ 0.001 in left side and *F* = 8.652, *p* ≤ 0.001 in right side; for MF, *F* = 6.312, *p*=0.002 in left side and *F* = 1.975, *p*=0.143 in right side). The independent *t*-test showed that both TrA and MF muscle thickness of the monitoring group is much greater than of the control group in 8 weeks, indicating that monitoring by RUSI and surface EMG was effective in core stability training.

**Figure 2:**
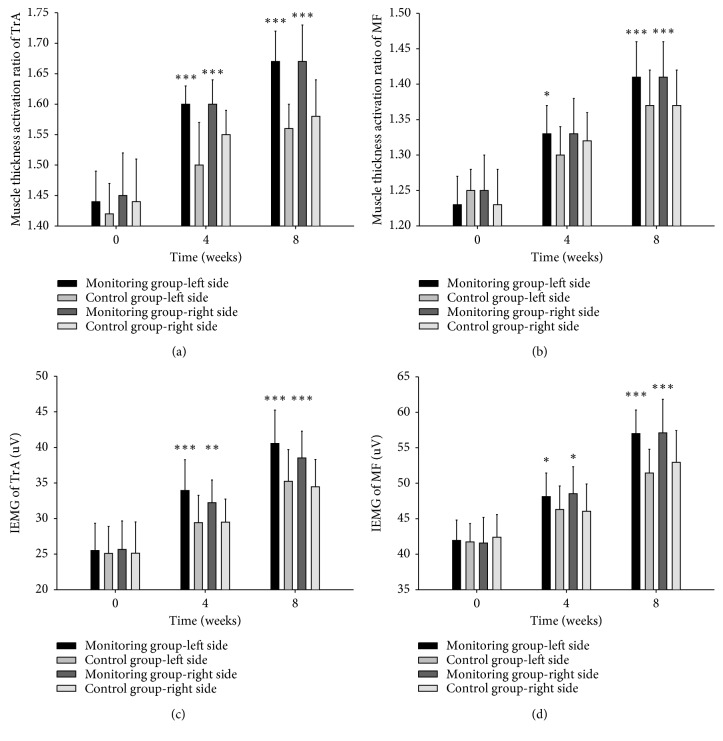
Comparison of the effect on local core muscles between the monitoring group and control group in 0, 4, and 8 weeks. (a) Comparison of the muscle thickness activation ratio of TrA between the monitoring group and control group in 0, 4, and 8 weeks. Activation ratio = contraction thickness/relax thickness. (b) Comparison of the muscle thickness activation ratio of MF between the monitoring group and control group in 0, 4, and 8 weeks. Activation ratio = contraction thickness/relax thickness. (c) Comparison of the IEMG of TrA between the monitoring group and control group in 0, 4, and 8 weeks. (d) Comparison of the IEMG of MF between the monitoring group and control group in 0, 4, and 8 weeks Values are means ± s.e.m. *p* values were calculated by the two-tailed Student's *t*-test. ^*∗∗∗*^*p* value < 0.001; ^*∗∗*^*p* value < 0.01; ^*∗*^*p* value < 0.05.

Meanwhile, the paired *t*-test showed that both TrA and MF muscle thickness after 8 weeks' exercise were significantly greater than baseline (*p* < 0.05), indicating that the core stability training is effective in improving the thickness of the local core muscles (Tables 2 and 3; Figures 2(a) and 2(b)).

### 3.3. EMG Data on Muscle Activity

All variables meet the normal distribution. Sphericity assumptions in both sides were identified by the test (*p*=0.698 (left) and *p*=0.942 (right) for TrA; *p*=0.106 (left) and *p*=0.516 (right) for MF), and the ANOVA for repeated measures can be used to perform the data analysis. The results show that the interaction between treatment effects and time effects was significant (for TrA, *F* = 10.876, *p* ≤ 0.001 in left side and *F* = 3.986, *p*=0.021 in right side; for MF, *F* = 13.243, *p*=0.005 in left side and *F* = 9.205, *p* ≤ 0.001 in right side). The independent *t*-test showed that the change of activation of TrA and MF in the monitoring group is greater than that in the control group.

Meanwhile, the mean EMG amplitudes of the TrA and MF were significantly increased after intervention, which shows that deep core muscle activation was effectively promoted following the core stability training (Tables 4 and 5; Figures 2(c) and 2(d)).

## 4. Discussion

Nowadays, an increasing number of pain rehabilitation strategy has been demonstrated effective to treat low back pain (LBP), but the results are always barely satisfactory [23, 24]. It was reported that muscle force imbalance may lead to kinetic instability of the spine, while the weakness of MF and TrA contributes notably to the development of LBP. Moreover, our previous study has shown that decreased cross-sectional area (CSA) of MF is correlated with chronic LBP. Here, we preliminarily verified the effectiveness of core stability training on the restoration of TrA and MF activation and strength. This training targeting local core muscles might be an alternative strategy for LBP management.

The activation ratio formula, which divides muscle thickness in a contraction state by muscle thickness at rest, has shown to be a more reliable method of using the thickness measures by RUSI [25, 26] as a function of activation for TrA and MF. By normalizing the size during a contracted state to the resting size, clinicians can determine the ability to activate the TrA and MF. However, this formula does not take into account the ability to isolate the TrA or MF without evoking a contraction of the superficial core muscles wall. Fortunately, the use of surface EMG can cover the shortage of RUSI. It was reported that the integrated EMG value determined by surface EMG showed positive correlation with muscle force and muscular tension [14], but the condition of deeper muscles may play roles in evaluating surface EMG signal of superficial core muscles during training, which means it is necessary for RUSI to avoid weakness of surface EMG. To sum up, the chance of error decreases when RUSI and EMG monitoring are applied simultaneously.

Compared with the control group, the monitoring group demonstrated better performance during training as expected. Effective core stability training which targeted specific core muscles can increase the number of contraction units and strengthen muscle force [27, 28], which is shown as the characteristic parameters of muscle thickness by ultrasound imaging and surface electromyography. The key of specific training is to ensure the target muscle contracts in normal patterns. The effectiveness of core stability training depends on the contraction sequence and relax patterns [11, 29]. Compensation activity during the process of training will affect the effectiveness of the training. During training targeting local core muscles, the activities of rectus abdominis and erector spinae were not allowed. Thus, the monitoring of these muscles was used to provide real-time training feedback to participants and guarantee the correct training. Based on this research, TrA and MF in both sides showed higher activation ratio and IEMG values than the control group without monitoring during training, while the global core muscles have no change (data not shown). We found that participants benefited greatly from the core stability training monitored by RUSI and surface EMG and reduced compensatory motion. Moreover, our findings taken together with previous studies corroborate the fact that specific monitoring training facilitating selective control of the TrA and MF independently of the other abdominal and back muscles, which can be monitored by RUSI and surface EMG, can be more beneficial for core stability than global, whole-body exercise programs.

In the beginning of this study, obvious compensatory action appears in surface EMG (data not shown) and the thickness of erector spinae or rectus abdominis increased significantly in RUSI. After long-term monitoring training, these activities vanished gradually in the same participant, and this phenomenon matched the improvement of IEMG of target muscles. In brief, through real-time monitoring during training, the generation of compensatory actions can be prevented. Besides, the training intensity and exercise time can be guaranteed to meet the training objectives strictly according to the rehabilitation plan.

This study examined the effect of core stability training on the change in muscle thickness and activation ratio in healthy individuals. Other issues to be considered in future research could be the type and timing of the core stability training on local core muscles of individuals with LBP. Whether the effect on healthy individuals is similar to that in patients is still need to be found out.

Together, this study is an important empirical evidence which investigates the intensive effects of core stability training monitored by RUSI and surface EMG on local core muscles of healthy people. The training monitored by RUSI and surface EMG can markedly enhance TrA and MF thickness activation ratio and IEMG in healthy human, providing an effective method of core stability training for LBP patients.

## Figures and Tables

**Table 1:** Demographic characteristics data of participants (mean ± SD).

	Monitoring group	Control group	*p* value
Age (y)	22.33 ± 1.47	22.30 ± 1.56	0.932
Height (m)	1.73 ± 0.08	1.71 ± 0.07	0.595
Weight (kg)	63.87 ± 8.18	62.77 ± 7.78	0.344
BMI (kg/m^2^)	21.30 ± 2.17	21.37 ± 1.71	0.901

**Table 2:** Comparison of the muscle thickness activation ratio of TrA between the monitoring group and control group in 0, 4, and 8 weeks (mean ± SD).

		Monitoring group	Control group	Between-group *p* value
Left	Baseline	1.44 ± 0.05	1.42 ± 0.05	0.251
	4 weeks	1.60 ± 0.03^*∗*^	1.50 ± 0.07^*∗*^	≤0.001
	8 weeks	1.67 ± 0.05^*∗*^	1.56 ± 0.04^*∗*^	≤0.001
Intragroup *p* value (baseline vs 8 weeks)		≤0.001	≤0.001	
Mauchly's sphericity test *W* = 0.970 (*p*=0.424)				

Right	Baseline	1.45 ± 0.07	1.44 ± 0.07	0.552
	4 weeks	1.60 ± 0.04^*∗*^	1.55 ± 0.04^*∗*^	≤0.001
	8 weeks	1.67 ± 0.06^*∗*^	1.58 ± 0.06^*∗*^	≤0.001
Intragroup *p* value (baseline vs 8 weeks)		≤0.001	≤0.001	
Mauchly's sphericity test *W* = 0.939 (*p*=0.165)				

Activation ratio = contraction thickness/relax thickness. ^*∗*^Compared with baseline, *p* < 0.05.

**Table 3:** Comparison of the muscle thickness activation ratio of MF between the monitoring group and control group in 0, 4, and 8 weeks (mean ± SD).

		Monitoring group	Control group	Between-group *p* value
Left	Baseline	1.23 ± 0.04	1.25 ± 0.03	0.328
	4 weeks	1.33 ± 0.04^*∗*^	1.30 ± 0.04^*∗*^	0.013
	8 weeks	1.41 ± 0.05^*∗*^	1.37 ± 0.05^*∗*^	≤0.001
Intragroup *p* value (baseline vs 8 weeks)		≤0.001	≤0.001	
Mauchly's sphericity test *W* = 0.986 (*p*=0.660)				

Right	Baseline	1.25 ± 0.05	1.23 ± 0.05	0.117
	4 weeks	1.33 ± 0.05^*∗*^	1.32 ± 0.04^*∗*^	0.645
	8 weeks	1.41 ± 0.05^*∗*^	1.37 ± 0.05^*∗*^	0.002
Intragroup *p* value (baseline vs 8 weeks)		≤0.001	≤0.001	
Mauchly's sphericity test *W* = 0.930 (*p*=0.127)				

Activation ratio = contraction thickness/relax thickness. ^*∗*^Compared with baseline, *p* < 0.05.

**Table 4:** Comparison of the IEMG of TrA between the monitoring group and control group in 0, 4, and 8 weeks (uV, mean ± SD).

		Monitoring group	Control group	Between-group *p* value
Left	Baseline	25.52 ± 3.82	25.11 ± 3.78	0.680
	4 weeks	33.94 ± 4.33^*∗*^	29.43 ± 3.83^*∗*^	≤0.001
	8 weeks	40.55 ± 4.69^*∗*^	35.24 ± 4.46^*∗*^	≤0.001
Intragroup *p* value (baseline vs 8 weeks)		≤0.001	≤0.001	
Mauchly's sphericity test *W* = 0.987 (*p*=0.698)				

Right	Baseline	25.67 ± 3.99	25.13 ± 4.40	0.617
	4 weeks	32.23 ± 3.19^*∗*^	29.50 ± 3.23^*∗*^	0.002
	8 weeks	38.52 ± 3.76^*∗*^	34.47 ± 3.84^*∗*^	≤0.001
Intragroup *p* value (baseline vs 8 weeks)		≤0.001	≤0.001	
Mauchly's sphericity test *W* = 0.998 (*p*=0.942)				

^*∗*^Compared with baseline, *p* < 0.05.

**Table 5:** Comparison of the IEMG of MF between the monitoring group and control group in 0, 4, and 8 weeks (uV, mean ± SD).

		Monitoring group	Control group	Between-group *p* value
Left	Baseline	41.94 ± 2.88	41.73 ± 2.60	0.769
	4 weeks	48.12 ± 3.32^*∗*^	46.30 ± 3.32^*∗*^	0.038
	8 weeks	57.00 ± 3.32^*∗*^	51.45 ± 3.34^*∗*^	≤0.001
Intragroup *p* value (baseline vs 8 weeks)		≤0.001	≤0.001	
Mauchly's sphericity test *W* = 0.924 (*p*=0.106)				

Right	Baseline	41.58 ± 3.62	42.40 ± 3.19	0.355
	4 weeks	48.53 ± 3.80^*∗*^	46.05 ± 3.85^*∗*^	0.015
	8 weeks	57.10 ± 4.72^*∗*^	52.95 ± 4.48^*∗*^	≤0.001
Intragroup *p* value (baseline vs 8 weeks)		≤0.001	≤0.001	
Mauchly's sphericity test *W* = 0.977 (*p*=0.516)				

^*∗*^Compared with baseline, *p* < 0.05.

## Data Availability

This study was qualified and registered in the Chinese Clinical Trial Registry (http://www.chictr.org.cn) by the authors, and all the data used to support the findings of this study, including RUSI muscle thickness and surface EMG characteristic, have been deposited in the Clinical Trial Management Public Platform (http://www.medresman.org) as ChiCTR1800014609.
